# Basic fibroblast growth factor promotes VEGF-C-dependent lymphangiogenesis via inhibition of miR-381 in human chondrosarcoma cells

**DOI:** 10.18632/oncotarget.9570

**Published:** 2016-05-24

**Authors:** Huey-En Tzeng, An-Chen Chang, Chun-Hao Tsai, Shih-Wei Wang, Chih-Hsin Tang

**Affiliations:** ^1^ Graduate Institute of Clinical Medical Science, China Medical University, Taichung, Taiwan; ^2^ Division of Hematology and Oncology, Department of Internal Medicine, China Medical University Hospital, Taichung, Taiwan; ^3^ Institute of Biomedical Sciences, National Chung Hsing University, Taichung, Taiwan; ^4^ Department of Orthopedic Surgery, China Medical University Hospital, Taichung, Taiwan; ^5^ Department of Medicine, Mackay Medical College, New Taipei City, Taiwan; ^6^ Graduate Institute of Basic Medical Science, China Medical University, Taichung, Taiwan; ^7^ Department of Pharmacology, School of Medicine, China Medical University, Taichung, Taiwan; ^8^ Department of Biotechnology, College of Health Science, Asia University, Taichung, Taiwan

**Keywords:** bFGF, chondrosarcoma, lymphangiogenesis, VEGF-C, miR-381

## Abstract

A chondrosarcoma is a common, primary malignant bone tumor that can grow to destroy the bone, produce fractures and develop soft tissue masses. Left untreated, chondrosarcomas metastasize through the vascular system to the lungs and ultimately lead to large metastatic deposits of the malignant cartilage taking over lung volume and function. Vascular endothelial growth factor (VEGF)-C has been implicated in tumor-induced lymphangiogenesis and elevated expression of VEGF-C has been found to correlate with cancer metastasis. bFGF (basic fibroblast growth factor), a secreted cytokine, regulates biological activity, including angiogenesis and metastasis. We have previously reported on the important role of bFGF in angiogenesis in chondrosarcomas. However, the effect of bFGF in VEGF-C regulation and lymphangiogenesis in chondrosarcomas is poorly understood. In this investigation, we demonstrate a correlation exists between bFGF and VEGF-C in tissue specimens from patients with chondrosarcomas. To examine the lymphangiogenic effect of bFGF, we used human lymphatic endothelial cells (LECs) to mimic lymphatic vessel formation. We found that bFGF-treated chondrosarcomas promoted LEC tube formation and cell migration. In addition, bFGF knockdown inhibited lymphangiogenesis *in vitro* and *in vivo*. We also found that bFGF-induced VEGF-C is mediated by the platelet-derived growth factor receptor (PDGFR) and c-Src signaling pathway. Furthermore, bFGF inhibited microRNA-381 expression via the PDGFR and c-Src cascade. Our study is the first to describe the mechanism of bFGF-promoted lymphangiogenesis by upregulating VEGF-C expression in chondrosarcomas. Thus, bFGF could serve as a therapeutic target in chondrosarcoma metastasis and lymphangiogenesis.

## INTRODUCTION

Chondrosarcomas are malignant cartilaginous tumors that account for around 26% of all primary malignant bone tumors. Chondrosarcomas usually occur in males aged between 10 and 80 years, and are most commonly found in the scapula, sternum, ribs, or pelvis [1, 2]. It has been reported that chondrosarcomas can easily metastasize to other organs, such as the lung and liver [3]. Distant metastasis means poor prognosis. Surgical resection remains the primary mode of therapy for chondrosarcomas. The lack of effective adjuvant therapies means that the development of novel therapeutic strategies is very important for chondrosarcoma metastasis [4, 5].

The metastatic spread of tumor cells is associated with resistance to conventional therapy and is the leading cause of death for cancer patients [6, 7]. Tumor metastasis is promoted by many processes in cell functions, such as proliferation [8] and migration of tumor cells [9], invasion [10], tumor-associated angiogenesis [11] and lymphangiogenesis [12]. Of all these processes, lymphangiogenesis is a key step during tumor metastasis. Preclinical evidence suggests that inhibiting cancer-mediated lymphangiogenesis may block the spread of cancer [13]. The development of new therapeutic strategies in the treatment of cancer depend on the identification of mechanisms underlying tumor lymphangiogenesis. Vascular endothelial growth factor (VEGF)-C is the best-characterized lymphangiogenic factor and reportedly plays a crucial role in lymphangiogenesis and lymphatic metastasis [14, 15]. In addition, many observations have noted that VEGF-C plays a pivotal role in various types of human cancers, such as colon [16], colorectal [17], acute myeloid leukemia [18], and lung cancer [19].

Basic fibroblast growth factor/fibroblast growth factor 2 (bFGF/FGF-2), a secreted cytokine, encodes heparin-binding proteins with growth, proliferation, differentiation, anti-apoptotic and angiogenic activity [20]. Its correlation with progression is certified in many cancers, e.g., oral squamous cell carcinoma, and correlates with lymph node metastasis and prognosis [21]. Expression of bFGF is associated with tumor recurrence and reduced survival after surgical resection of oesophageal cancer [22]. In chondrosarcoma, we have previously reported that bFGF increases VEGF-A expression and subsequently promotes endothelial progenitor cell-primed angiogenesis [23], implying that bFGF is involved in the metastatic process of chondrosarcoma.

Several studies have focused on the role of microRNAs (miRNAs) in cancer progression and metastasis [24, 25]. miRNAs influence numerous cancer-relevant processes such as proliferation, apoptosis, migration, invasion, angiogenesis, and lymphangiogenesis [26]. miRNAs are short, noncoding RNA molecules, with an average length of about 18 to 22 nucleotides. They bind to the 3′ untranslated region (3′-UTR) of mRNA through complementary base pairing, resulting in mRNA degradation or translation inhibition [27]. miRNAs have been reported to inhibit tumor lymphangiogenesis through dysregulation of the miR/VEGF-C axis [28]. Investigations have documented that miR-128 suppresses human non-small cell lung cancer lymphangiogenesis by directly inhibiting VEGF-C expression [29], while overexpression of miR-206 attenuates VEGF-C levels and lymphangiogenesis in pancreatic adenocarcinoma [30]. However, the role of miRNA in regulating bFGF-mediated VEGF-C expression in chondrosarcoma is largely unknown. In the present study, we found that bFGF promotes VEGF-C expression in chondrosarcoma and subsequently enhances lymphangiogenesis of lymphatic endothelial cells (LECs). In addition, miR-381 is negatively regulated by bFGF through transactivating the platelet-derived growth factor receptor (PDGFR)/c-Src signaling pathway.

## RESULTS

### Clinical significance of bFGF and VEGF-C expression in specimens from patients with chondrosarcomas

**Figure 1:**
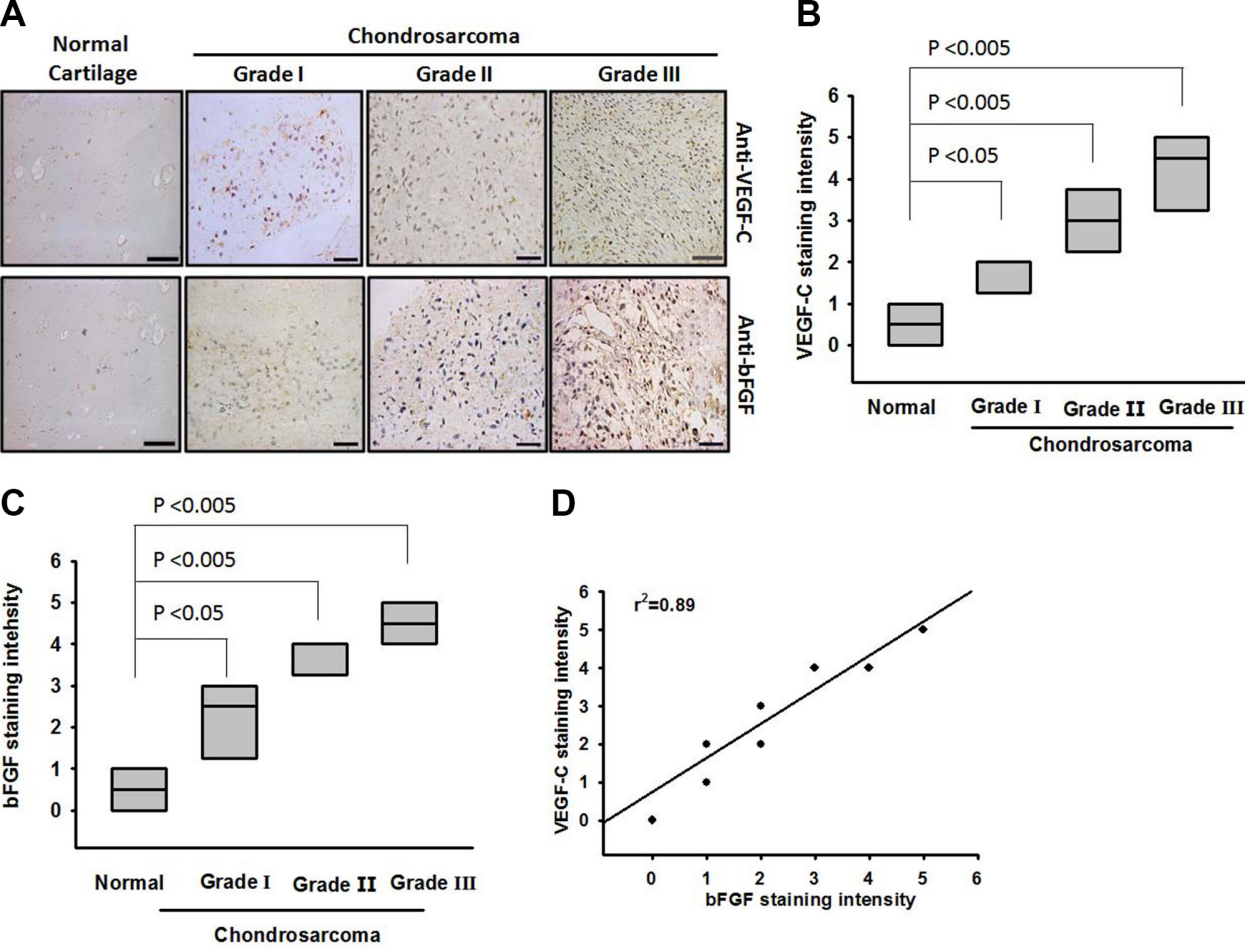
Clinical significance of bFGF and VEGF-C in specimens from patients with chondrosarcoma Tumor specimens were immunostained with anti-VEGF-C antibody. Staining intensity was scored 1–5. (**A**) IHC photographs (Scar bar = 50 μm). The quantitated results are shown in (**B** and **C**). (**D**) Correlation between bFGF, VEGF-C, and chondrosarcoma clinical grades. Data are expressed as the mean ± SEM.

Our previous study indicated that bFGF is associated with chondrosarcoma progression and angiogenesis [23]. We have also previously reported finding a higher expression of bFGF in chondrosarcoma patients compared with normal cartilage [23]. To examine the role of bFGF in the lymphangiogenesis of chondrosarcoma, we analyzed the VEGF-C expression profile in specimens from patients with chondrosarcomas. The results indicated that VEGF-C expression was higher in tumor specimens than in normal tissues and correlated with tumor stage (Figure 1A and 1B). In addition, the mRNA expression of bFGF and VEGF-C in chondrosarcoma patients was higher than that in normal cartilage (Supplementary Figure S1). The quantitative data also showed that bFGF expression was correlated with VEGF-C expression in human chondrosarcoma specimens (Figure 1C), indicating that bFGF is associated with VEGF-C expression and tumor stage in patients with chondrosarcomas.

### Involvement of VEGF-C expression in bFGF–directed lymphangiogenesis of chondrosarcoma

**Figure 2:**
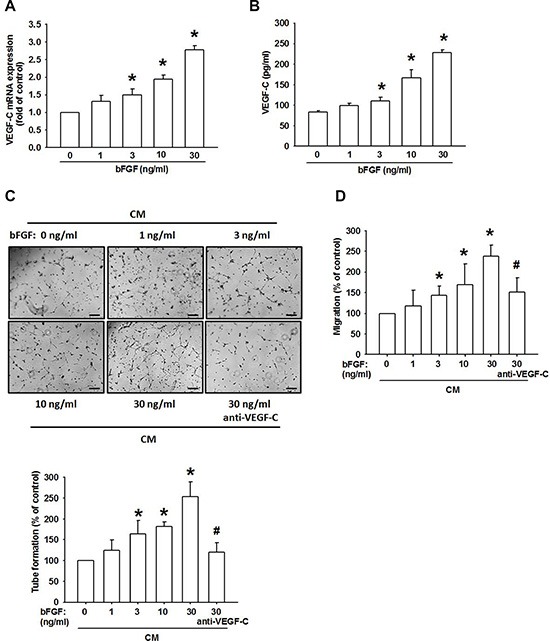
bFGF promotes the lymphangiogenesis through upregulation of VEGF-C in chondrosarcoma cells (**A** and **B**) JJ012 cells were incubated with bFGF (1–100 ng/mL) for 24 h, VEGF-C expression was measured by qPCR and ELISA (*n* = 6–8). (**C** and **D**) JJ012 cells were incubated with bFGF (1–30 ng/mL) for 24 h, or pretreated for 30 min with IgG control antibody or VEGF-C antibody (1 μg/mL), followed by stimulation with bFGF (30 ng/mL) for 24 h. Medium was collected as CM, then applied to LECs for 24 h. Capillary-like structure formation and *in vitro* cell migration in LECs were examined by tube formation and the Transwell assay (Scar bar = 100 μm) (*n* = 6–8). Data are expressed as the mean ± SEM: *P < 0.05 compared to controls; ^#^*P* < 0.05 compared to the bFGF-treated group.

VEGF-C has been reported to mediate lymphangiogenesis of human cancer cells [31]. We therefore examined whether VEGF-C is involved in bFGF-induced lymphangiogenesis of chondrosarcoma cells. Incubation of a chondrosarcoma cell line (JJ012 cells) increased VEGF-C mRNA expression and protein secretion (Figure 2A and 2B). Besides, we previous also indicated that bFGF increased VEGF-A expression in chondrosarcoma [23]. Lymphangiogenesis involves proliferation, migration, and tube formation of LECs to form new lymph vessels [32]. We then examined whether bFGF-dependent VEGF-C expression induced lymphangiogenesis by using an *in vitro* LEC model. Incubation of LECs with conditioned medium (CM) from bFGF-treated JJ012 cells dramatically enhanced LEC migration and tube formation (Figure 2C and 2D). On the other hand, bFGF-stimulated chondrosarcoma CM also promoted tube formation in endothelial cells [23]. Conversely, VEGF-C mAb abolished bFGF-mediated LEC migration and tube formation (Figure 2C and 2D), implying that bFGF promotes lymphangiogenesis through a VEGF-C-dependent pathway.

### bFGF promotes VEGF-C expression in chondrosarcoma cells through the PDGFR/c-Src pathway

**Figure 3:**
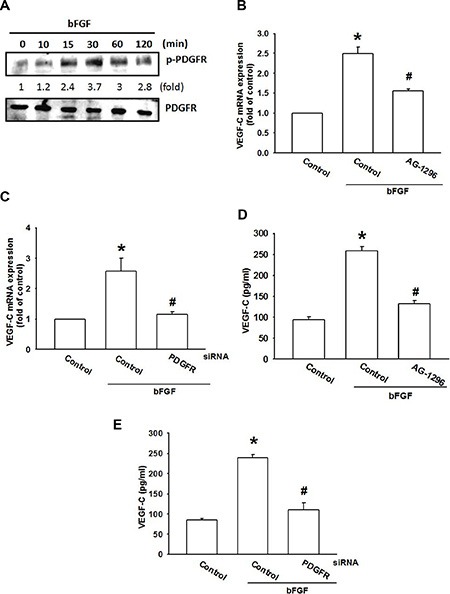
The PDGFR signaling pathway is involved in bFGF-induced VEGF-C expression (**A**) JJ012 cells were incubated with bFGF (30 ng/mL) for the indicated time intervals; PDGFR phosphorylation was examined by western blotting (*n* = 5). (**B**–**E**) JJ012 cells were pretreated for 30 min with AG-1296 (3 μM) or transfected with PDGFR siRNA for 24 h, followed by stimulation with bFGF (30 ng/mL) for 24 h. VEGF-C expression was examined by qPCR and ELISA (*n* = 5–7). Data are expressed as the mean ± SEM: *P < 0.05 compared to controls; ^#^*P* < 0.05 compared to the bFGF-treated group.

**Figure 4:**
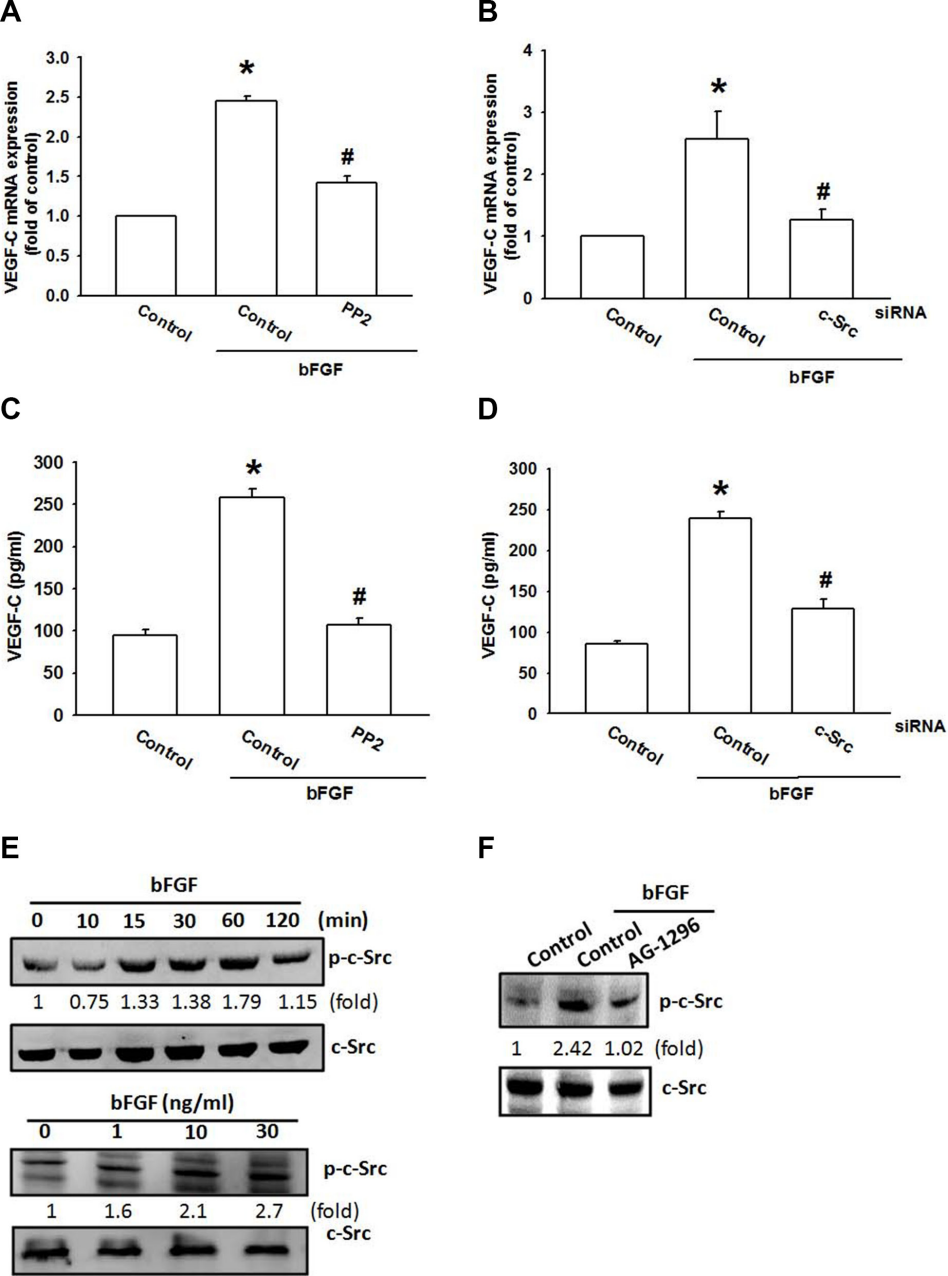
c-Src activation is involved in bFGF-induced VEGF-C expression (**A**–**D**) JJ012 cells were pretreated for 30 min with PP2 (3 μM) or transfected with c-Src siRNA for 24 h, followed by stimulation with bFGF (30 ng/mL) for 24 h. VEGF-C expression was examined by qPCR and ELISA (*n* = 6–8). (**E**) JJ012 cells were incubated with bFGF (30 ng/mL) for the indicated time intervals or indicated concentrations for 30 min; c-Src phosphorylation was examined by western blotting (*n* = 5). (**F**) JJ012 cells were pretreated for 30 min with PP2 (3 μM), followed by stimulation with bFGF (30 ng/mL) for 24 h; c-Src phosphorylation was examined by western blotting (*n* = 5). Data are expressed as the mean ± SEM: *P < 0.05 compared to controls; ^#^*P* < 0.05 compared to the bFGF-treated group.

bFGF has been found to enhance cell migration through PDGFR activation [33]. We therefore analyzed PDGFR signaling in bFGF-increased VEGF-C expression in chondrosarcoma cells. Therefore, we examined PDGFR activation, and found that bFGF increased PDGFR phosphorylation in a time-dependent manner (Figure 3A). In addition, treatment with a PDGFR-specific inhibitor (AG-1296) or transfection with PDGFR siRNA diminished bFGF-increased VEGF-C expression (Figure 3B–3E). Thus, bFGF appears to act through the PDGFR signaling pathway to promote VEGF-C expression in human chondrosarcoma cells.

c-Src tyrosine kinase is a downstream molecule in PDGFR signaling [34]. We next examined whether PDGFR-dependent c-Src activation is involved in bFGF-induced VEGF-C expression. Pretreatment of cells with a c-Src inhibitor (PP2) or transfection of cells with c-Src siRNA abolished bFGF-induced VEGF-C expression (Figure 4A–4D). c-Src phosphorylation was increased after bFGF treatment time and dose-dependently (Figure 4E). Conversely, pretreatment with AG-1296 markedly diminished bFGF-induced c-Src phosphorylation (Figure 4F). Based on these results, it appears that bFGF acts through the PDGFR and c-Src pathways to enhance VEGF-C expression in chondrosarcoma cells.

### bFGF promotes VEGF-C production via inhibition of miR-381 expression

**Figure 5:**
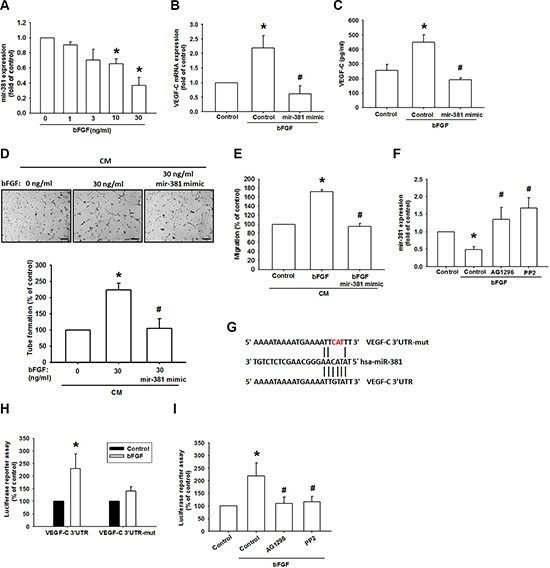
bFGF promotes VEGF-C via downregulation of miR-381 (**A**) JJ012 cells were incubated with bFGF (0–30 ng/mL) for 24 h; miR-381 expression was examined by qPCR (*n* = 6). (**B** and **C**) JJ012 cells were transfected with miRNA control or miR-381 mimic for 24 h and stimulated with bFGF (30 ng/mL) for 24 h. VEGF-C expression was examined by qPCR and ELISA (*n* = 5–7). (**D** and **E**) Medium was collected as CM, then applied to LECs for 24 h; capillary-like structure formation and *in vitro* cell migration in LECs was examined by tube formation and the Transwell assay (Scar bar = 100 μm) (*n* = 5–7). (**F**) Cells were pretreated for 30 min with AG-1296 or PP2 and stimulated with bFGF (30 ng/mL) for 24 h. miR-381 expression was examined by qPCR (*n* = 5). (**G**) Schematic 3′UTR representation of the human VEGF-C containing miR-381 binding site. (**H**) JJ012 cells were transfected with the wt-VEGFC-3′UTR or mt-VEGFC-3′UTR plasmids for 24 h and stimulated with bFGF (30 ng/mL) for 24 h; relative luciferase/renilla activities were measured as described in the Methods section (*n* = 5). (**I**) JJ012 cells were pretreated for 30 min with AG-1296 or PP2 and stimulated with bFGF for 24 h. wt-VEGFC-3′UTR relative luciferase/renilla activities were measured as described in the Methods section (*n* = 5). Data are expressed as the mean ± SEM: *P < 0.05 compared to controls; ^#^*P* < 0.05 compared to the bFGF-treated group.

miRNAs are important regulators in tumor angiogenesis, which makes them promising therapeutic targets [35]. miRNA target prediction using open-source software (www.TargetScan.org and www.microrna.org) revealed that the 3′UTR region of VEGF-C mRNA harbors potential binding sites for miR-381. Exogenous bFGF reduced miR-381 expression in a concentration-dependent manner (Figure 5A). To explore miR-381 involvement in bFGF-induced VEGF-C and lymphangiogenesis, miR-381 mimic was used; transfection with miR-381 mimic diminished bFGF-induced VEGF-C expression (Figure 5B and 5C). Conversely, miR-381 mimic also diminished bFGF-promoted LEC migration and tube formation (Figure 5D and 5E). Furthermore, AG-1296 and PP2 reversed bFGF-inhibited miR-381 expression (Figure 5F), indicating that bFGF promotes VEGF-C expression and lymphangiogenesis by suppressing miR-381 expression via the PDGFR and c-Src pathways.

To learn whether miR-381 regulates the 3′UTR region of VEGF-C, we constructed luciferase reporter vectors harboring the wild-type 3′UTR region of VEGF-C mRNA (wt-VEGFC-3′UTR) and vector containing mismatches in the predicted miR-381 binding site (mt-VEGFC-3′UTR) (Figure 5G). The results show that bFGF increased luciferase activity in the wt-VEGFC-3′UTR plasmid but not in the mt-VEGFC-3′UTR plasmid (Figure 5H). In addition, treatment with AG-1296 and PP2 diminished bFGF-promoted wt-VEGFC-3′UTR luciferase activity (Figure 5I). These data suggest that miR-381 directly represses VEGF-C protein expression via binding to the 3′UTR region of the human *VEGF-C* gene through PDGFR and c-Src signaling.

### Inhibiting bFGF expression suppresses lymphangiogenesis *in vivo*

**Figure 6:**
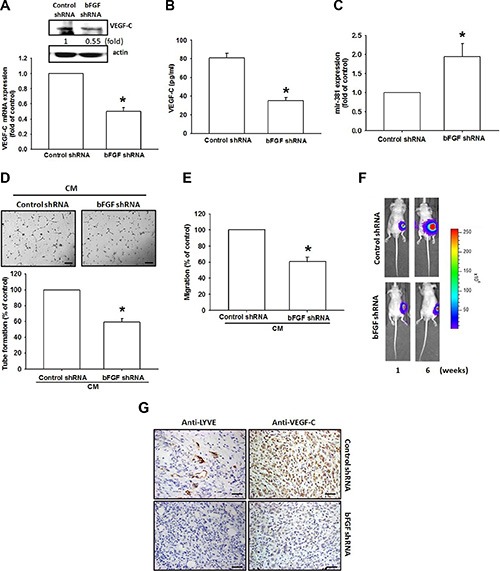
bFGF knockdown in chondrosarcomas decreases lymphangiogenesis *in vivo* (**A**–**C**) VEGF-C and mR-381 expression in JJ012/control shRNA and JJ012/bFGF shRNAs were examined by qPCR, Western blotting and ELISA (*n* = 3). (**D** and **E**) LECs were incubated with CM collected from JJ012/control shRNAs or JJ012/bFGF shRNAs for 24 h; tube formation or cell migration was examined by photographs or Transwell assay (*n* = 5). (**F**) JJ012/control shRNA and JJ012/bFGF shRNA cells were mixed with Matrigel and injected into the flanks of mice, and tumors were monitored by bioluminescence imaging. (**G**) After 42 days, the tumors were embedded in paraffin and sections were immunostained using integrin LYEC and VEGF-C antibodies (Scar bar = 50 μm) (*n* = 6). Data are expressed as the mean ± SEM: *P < 0.05 compared to controls; ^#^*P* < 0.05 compared to the bFGF-treated group.

Here, we found that bFGF promoted VEGF-C expression in chondrosarcomas and enhanced LEC lymphangiogenesis. It is critical to pinpoint the role of bFGF *in vivo*. Previously, we established JJ012 cells stably expressing bFGF shRNA, in which we found that the expression of bFGF was decreased in bFGF shRNA stable clones [23]. In this study, bFGF knockdown significantly reduced the expression of VEGF-C (Figure 6A and 6B) and increased miR-381 expression (Figure 6C). CM collected from JJ012/control shRNAs promoted LEC cell migration and tube formation, but this activity was decreased during incubation with CM collected from JJ012/bFGF shRNA (Figure 6D and 6E). We also previously found that bFGF knockdown reduced tumor growth in mice compared with the JJ012/control shRNA group [23]. Here, we used IHC staining to examine the level of lymphangiogenesis. Analysis revealed that bFGF knockdown impedes the expression of lymphatic markers LYEC and VEGF-C (Figure 6F and 6G) and inhibits lymphangiogenesis *in vivo*.

## DISCUSSION

Chondrosarcoma is estimated to account for 3.6% of the annual incidence of all primary bone malignancies in the USA, after multiple myeloma and osteogenic sarcoma [36]. Distant metastatic potential of chondrosarcoma has already been reported [3, 4]. Lymphangiogenesis is one of the major routes for tumor invasion and metastasis. VEGF-C is a key modulator in tumor lymphangiogenesis and metastasis, so is therefore a potential target for preventing tumor lymphatic metastasis. The effect of bFGF on angiogenesis in chondrosarcomas has been discussed previously [23]. Here, we provide novel insights into the role of bFGF in lymphangiogenesis. We report that a high level of bFGF expression correlates strongly with VEGF-C expression and tumor stage in chondrosarcoma patients. Our data show that bFGF promotes VEGF-C expression and increases lymphangiogenesis by downregulating miR-381 through the PDGFR and Akt signaling pathways, rendering bFGF as a novel target for chondrosarcoma lymphangiogenesis.

LECs are associated with the induction and modulation of VEGF-C during tumor metastasis [37], and lymphangiogenesis has recently become a possible therapeutic target for patients with chondrosarcomas [38]. However, it remains unclear as to whether LECs are involved with the regulation and mechanistic functions of bFGF in chondrosarcoma. Growing evidence indicates that LECs are associated with abnormal lymphangiogenesis via the induction and modulation of VEGF-C [39, 40]. In the present study, we found that CM from bFGF-treated chondrosarcoma cells increased LEC migration and tube formation, implying that bFGF enhances lymphangiogenesis in chondrosarcoma cells. Furthermore, VEGF-C mAb diminished bFGF-mediated lymphangiogenesis, indicating that bFGF promotes VEGF-C-dependent lymphangiogenesis in chondrosarcoma cells. Besides, whether other cytokines also involved in VEGF-C-dependent lymphangiogenesis are needs further examination.

Evidence indicates that PDGFR, a potential candidate signaling molecule, mediates bFGF-increased cell proliferation and migration [33]. Here, we report that both a PDGFR inhibitor and a siRNA antagonized bFGF-induced VEGF-C expression. Incubation of chondrosarcoma cells with bFGF promoted PDGFR phosphorylation, suggesting that PDGFR activation plays a crucial role in bFGF-increased VEGF-C production and lymphangiogenesis. Conversely, c-Src tyrosine kinase activation is an important downstream event of PDGFR signaling [41]. In the current study, inhibition of c-Src by a pharmacologic inhibitor or genetic siRNA reduced VEGF-C production. We also found that bFGF enhanced c-Src phosphorylation, and was inhibited by AG-1296. These findings show that PDGFR-dependent c-Src activation may have a key role in bFGF-increased VEGF-C expression and lymphangiogenesis.

The newly identified small noncoding miRNAs, a novel class of gene regulators, control gene expression by binding to the complementary 3′UTR sequences of target mRNA [42, 43]. miR-381 has been reported to inhibit migration and invasion by targeting inhibitor of differentiation 1 in human lung adenocarcinoma [44]; miR-381 is also indicated to be a negative regulator of cell growth in glioma [45], but its effect on VEGF-C expression is largely unknown. We found that exogenous bFGF reduced miR-381 expression. Co-transfection with miR-381 mimic reduced bFGF-induced VEGF-C expression as well as LEC migration and tube formation. In addition, we found that miR-381 directly represses VEGF-C protein expression through binding with the 3′UTR region of the human *VEGF-C* gene, thereby negatively regulating VEGF-C-mediated lymphangiogenesis. Furthermore, PDGFR and c-Src inhibitors reversed bFGF-mediated miR-381 expression as well as VEGF-C 3′UTR activity, implying that PDGFR and c-Src pathways are upstream molecules of bFGF-impaired miR-381 expression.

IHC results from clinical specimens from patients with chondrosarcomas demonstrated that bFGF and VEGF-C expression levels were positively correlated with tumor stage. In cellular and animal experiments, we indicate that bFGF promotes VEGF-C expression and lymphangiogenesis in chondrosarcomas. In addition, bFGF promotes VEGF-C expression and lymphangiogenesis by downregulating miR-381 expression via the PDGFR and c-Src signaling pathways. Thus, bFGF may be a new molecular therapeutic target in chondrosarcoma lymphangiogenesis and metastasis.

## MATERIALS AND METHODS

### Materials

Protein A/G beads; anti-mouse and anti-rabbit IgG-conjugated horseradish peroxidase; rabbit polyclonal antibodies specific for p-PDGFR, PDGFR, p-c-Src, and c-Src were purchased from Santa Cruz Biotechnology (Santa Cruz, CA, USA). Recombinant human bFGF was purchased from R&D Systems (Minneapolis, MN, USA). VEGF-C antibody was purchased from Abcam (Cambridge, MA, USA). Dulbecco's modified Eagle's medium (DMEM), F-12 medium, fetal bovine serum (FBS) and all other cell culture reagents were purchased from Gibco-BRL Life Technologies (Grand Island, NY, USA). ON-TARGETplus siRNAs were purchased from Dharmacon Research (Lafayette, CO, USA). miR-381 mimic, miRNA control, Lipofectamine 2000, and Trizol were purchased from Life Technologies (Carlsbad, CA, USA). All other chemicals were sourced from Sigma-Aldrich (St Louis, MO, USA).

### Cell culture

The human chondrosarcoma cell line JJ012 was donated by the laboratory of Dr. Sean P. Scully (University of Miami School of Medicine, Miami, FL, U.S.A.). Cells were cultured in complete medium containing DMEM/α-MEM with 10% (v/v) FBS supplement. The basal levels of bFGF in chondrosarcoma cell lines have been shown in Supplementary Figure S2.

Human telomerase-immortalized human dermal lymphatic endothelial cells (hTERT-HDLECs), an immortalized human LEC line, were purchased from Lonza (Walkersville, MD, USA). These immortalized human LECs are CD31-positive/podoplanin-positive, and retain their ability to uptake acetylated LDL and induce tube formation. Human LECs were grown in EGM-2MV BulletKit Medium consisting of EBM-2 basal medium plus SingleQuots kit (Lonza). Cells were seeded onto 1% gelatin-coated plastic ware and cultured at 37°C and 5% CO_2_. We obtained the cryopreserved human LECs line from Lonza as a secondary culture (passage 1) and maintained these cells according to the manufacturer's instructions. One set of cells underwent cell cycle analysis at passages 5 and 10 for experiments described below.

### Transwell migration assay

This process used transwell inserts (8-μm pore size; Costar, NY, USA) in 24-well plates. Chondrosarcoma cells were pretreated for 30 min with designated inhibitors or vehicle (0.1% dimethyl sulfoxide [DMSO]). Alternatively, chondrosarcoma cells were transfected with the indicated siRNAs for 24 h, and the conditioned medium (CM) was collected after 24 h. LECs were seeded in the upper transwell chamber and 300 μL of CM were placed in the lower chamber. After 20 h, the migratory cells were stained with crystal violet and counted under microscope.

### LECs tube formation

Matrigel (BD Biosciences, Bedford, MA, USA) was dissolved at 4°C, and 150 μL aliquots were added to each well of 48-well plates, which were incubated for 30 min at 37°C. LECs were resuspended at a density of 2 × 10^4^/100 μL in culture medium (50% EGM-MV2 medium and 50% chondrosarcoma cell CM) and added to the wells. After 6 h of incubation at 37°C, LEC tube formation was assessed by microscopy, and each well was photographed. The number of tube branches and total tube lengths were calculated using MacBiophotonics Image J software.

### Immunohistochemistry (IHC)

A human chondrosarcoma tissue array was purchased from Biomax (Rockville, MD; 18 cases for healthy cartilage, 39 cases for grade I chondrosarcoma, 17 cases for grade II chondrosarcoma, and 13 cases for grade III chondrosarcoma). The tissues were placed on glass slides, rehydrated, and incubated in 3% hydrogen peroxide to block endogenous peroxidase activity. After trypsinization, sections were blocked by incubation in 3% BSA in PBS. Primary monoclonal mouse anti-human VEGF-C antibody was applied to the slides at a dilution of 1:50 and incubated at 4°C overnight. Samples were washed with PBS 3 times, then treated with goat anti-mouse IgG biotin-labeled secondary antibody at a dilution of 1:50. Bound antibodies were detected with an ABC kit (Vector Laboratories). The slides were stained with chromogen diaminobenzidine, washed, counterstained with Delafield's hematoxylin, dehydrated, treated with xylene, and mounted. The staining intensity was evaluated as 0, 1+, 2+, 3+, 4+, and 5+ for no staining, very weak staining, weak staining, moderate staining, strong staining, and very strong staining, respectively, by two independent and blinded observers. IHC score was determined as the sum of the intensity score.

### ELISA assay

Cells (2 × 10^4^) were cultured in 24-well culture plates and incubated in a humidified incubator for 24 h at 37°C. After pretreatment with a pharmacologic inhibitor or transfection with siRNA, followed by stimulation with bFGF for 24 h, the medium was removed and stored at −80°C until assay. Concentrations of VEGF-C in the medium were assayed using the VEGF-C enzyme immunoassay kit (R&D Systems; Minneapolis, MN, USA), according to manufacturer's procedure.

### Western blot analysis

Cells were collected and lysed in cold RIPA buffer containing protein inhibitors. Proteins resolved by SDS-PAGE were transferred to Immobilon polyvinyldifluoride (PVDF) membrane. Blots were blocked with 4% BSA for 1 h at room temperature, then probed with rabbit anti-human antibodies against p-PDGFR, PDGFR, p-c-Src or c-Src (1:1000) for 1 h at room temperature. After three washes, blots were subsequently incubated with a donkey anti-rabbit peroxidase-conjugated secondary antibody (1:1000) for 1 h at room temperature and visualized by enhanced chemiluminescence, using Imagequant LAS 4000 (GE Healthcare, Pewaukee, WI) [46].

### Quantitative real-time polymerase chain reaction (qPCR)

Total RNA was extracted from JJ012 cells by using TRIzol reagent. The messenger RNA was reversely transcribed to complementary DNA by using MMLV RT kit, and qPCR was then performed by using Taqman assay kit. The qPCR analysis of miRNA expression was performed on StepOnePlus sequence detection system by using the TaqMan MicroRNA Reverse Transcription Kit and was normalized to U6 expression [47].

### Plasmid construction and luciferase reporter assay

Wild-type VEGF-C-3′-UTR was constructed into *pmirGLO* reporter vector between the *Nhe*I and *Xho*I cutting sites. The mutation of VEGF-C-3′-UTR was performed by Quickchange site directed kit (Stratagene; La Jolla, CA, USA) according to the manufacturer's instructions.

To analysis the 3′-UTR luciferase activity, the JJ012 cells were transfected with wt-VEGFC-3′UTR or mt-VEGFC-3′UTR luciferase plasmids. Cells were lysated after 24 hr transfection, cell lysated were harvested and detected using luciferase assay system (Promega; Madison, WI, USA).

### Statistics

Data are presented as the mean and standard error of the mean (SEM). Statistical analysis between 2 or more groups was performed using the Student's *t* test and multiple comparison was carried out using one-way analysis of variance with Bonferroni's post-hoc test. In all cases, *p* < 0.05 was considered significant.

## SUPPLEMENTARY MATERIALS FIGURES


