# Supplementary material for: Basic fibroblast growth factor promotes VEGF-C-dependent lymphangiogenesis via inhibition of miR-381 in human chondrosarcoma cells
Source: Oncotarget. 2016 May 24;7(25):38566–78. doi: 10.18632/oncotarget.9570 (PMC5122411; doi:10.18632/oncotarget.9570)
Supplement: Supplementary file 1 [file oncotarget-07-38566-s001.pdf]

Basic fibroblast growth factor promotes VEGF-C-dependent lymphangiogenesis via inhibition of miR-381 in human chondrosarcoma cells

Supplementary Materials

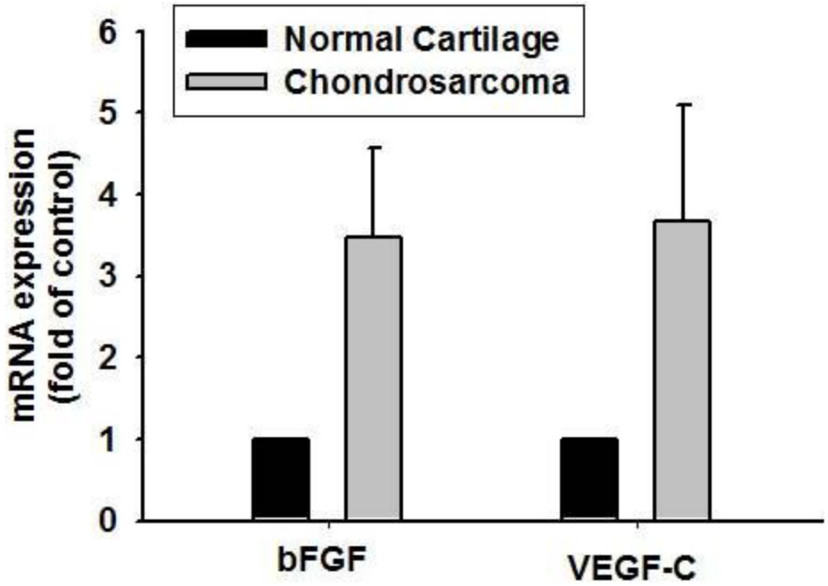

Supplementary Figure S1: bFGF and VEGF-C mRNA expression in normal cartilage and chondrosarcoma patients. The bFGF and VEGF-C mRNA expression in normal cartilage and chondrosarcoma patients was examined by qPCR. The quantitative results were expressed as mean ± SEM.

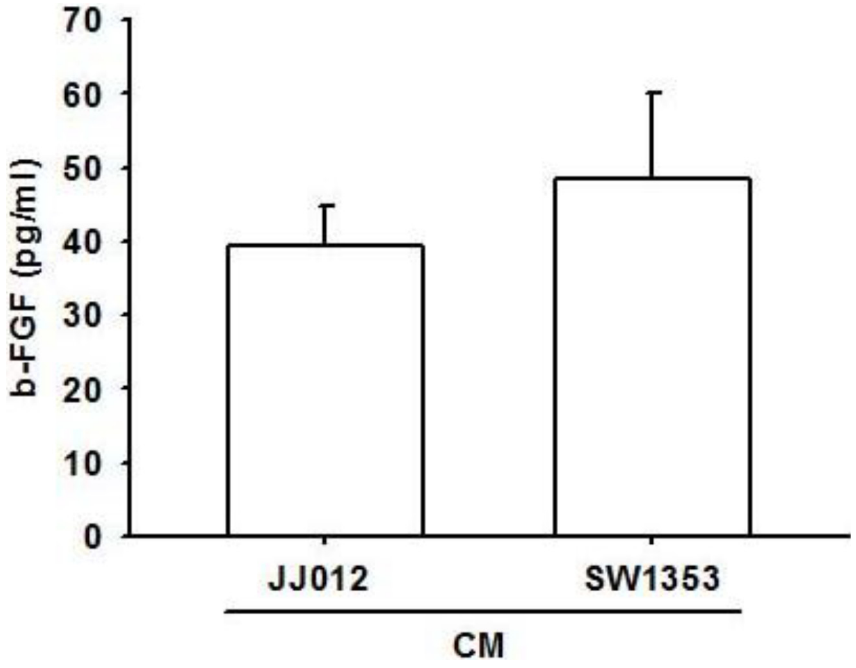

Supplementary Figure S2: The bFGF expression level in chondrosarcoma cell lines. JJ012 and SW1353 cells were cultured for 24 hr, the bFGF production was measured by ELISA. The quantitative results were expressed as mean ± SEM.
